# Cancer staging in individuals with a severe psychiatric illness: a cross-sectional study using population-based cancer registry data

**DOI:** 10.1186/s12885-020-06943-w

**Published:** 2020-05-27

**Authors:** Alyson L. Mahar, Paul Kurdyak, Timothy P. Hanna, Natalie G. Coburn, Patti A. Groome

**Affiliations:** 1 Department of Community Health Sciences, University of Manitoba, Rm 443 727 McDermot Ave, Winnipeg, MP R3E 3P5 Canada; 2 ICES, 2075 Bayview Ave, Toronto, ON M4N 3M5 Canada; 3 Centre for Addiction and Mental Health, T305 33 Russell Street, Toronto, ON M5S 2S1 Canada; 4 Division of Radiation Oncology, Department of Oncology, Queen’s University, 25 King St W, Kingston, ON K7L 3N6 Canada; 5 Odette Cancer Centre, Sunnybrook Health Sciences Centre, T2011 2075 Bayview Ave, Toronto, ON M4N 3M5 Canada; 6 Division of Cancer Care and Epidemiology, Queen’s University, 2nd Level 10 Stuart Street, Kingston, ON K7L 3N6 Canada

**Keywords:** Mental disorders, Neoplasms, Stage at diagnosis, cancer registry, unknown stage

## Abstract

**Background:**

Advanced cancer stage at diagnosis may explain high cancer mortality among patients with a severe psychiatric illness (SPI). Studies to date investigating advanced stage cancer at diagnosis as a potential explanation for high cancer mortality in individuals with a history of mental illness have been inconclusive. We examined the relationship between a SPI history and unknown cancer stage at diagnosis in colorectal cancer (CRC) patients.

**Methods:**

This was a population-based, cross-sectional study using linked administrative databases of CRC patients diagnosed between 01/04/2007 and 31/12/2012. Individuals who had a history of mental illness but did not meet the definition of a SPI were excluded. An SPI was measured in the 5 years prior to the cancer diagnosis and categorized as inpatient, outpatient or no SPI. Individuals with a best stage in Stage 0 to Stage IV were considered staged and absence of staging information was defined as unknown stage. The risk of unknown stage cancer was estimated using modified Poisson regression.

**Results:**

The final study cohort included 24,507 CRC patients. 258 (1.1%) individuals experienced a history of inpatient SPI and 482 (2.0%) experienced outpatient SPI. After adjusting for confounders, CRC patients with an inpatient or outpatient history of SPI were at greater risk of having missing TNM stage at diagnosis, compared to patients with no history of a mental illness (RR 1.45 (95% CI: 1.14–1.85) and RR1.17 (95% CI 0.95–1.43), respectively). The results did not change when alternate practices to assign SPI history using administrative data were used.

**Conclusions:**

Individuals with an SPI, especially those with a psychiatric admission, were more likely to have missing stage data compared to individuals without a history of a mental illness. Incomplete and low quality cancer staging data likely undermines the quality of cancer care following initial diagnosis. Understanding why patients with an SPI are missing this information is a critical first step to providing excellent care to this vulnerable population.

## Background

Advanced stage cancer at diagnosis has been suggested as a potential explanation for worse cancer case fatality in individuals with a history of mental illness compared with the rest of the cancer population [1–3]. Individuals with a severe psychiatric illness (SPI) history may be at increased risk for incurable stage cancer through a number of patient factors (e.g. low socioeconomic status), provider factors (e.g. diagnostic overshadowing) and healthcare system factors (fragmented healthcare) [4–6]. These factors are established risk factors for advanced stage of cancer at diagnosis [7–9].

Studies investigating advanced stage cancer at diagnosis as a potential explanation for high cancer mortality in individuals with a history of mental illness have been inconclusive [10, 11]. The exclusion of patients with missing stage data may contribute to these uncertain conclusions, particularly if patients with an SPI are overrepresented. For example, Chang et al. excluded almost 35% of the cohort who were missing stage data, leaving only 125 individuals (0.45%) in the SPI group [12]. They reported no difference in the proportion of missing data between individuals with a psychiatric illness and without [12]. In patients with colorectal cancer identified in the Surveillance, Epidemiology and End Results cancer registry, patients with a SPI history had a greater proportion of unknown stage of cancer at diagnosis (14%) compared to the general cancer population (6.2%) [13]. The proportion of patients with missing stage data was extremely high in persons with a psychotic disorder (22.9%) [13].

Incompleteness of diagnostic and staging data in medical charts and population-based cancer registries may result from limitations in the collection process when stage is known, or may reflect cases where clinical information on stage were not ascertained. Regardless, incomplete staging will almost certainly impact the subsequent quality of cancer care; either directly where stage is truly unknown, or indirectly through the creation and dissemination of inaccurate research findings. The objectives of this study were to compare the occurrence of missing data for a number of cancer registry diagnostic and staging variables and estimate the risk of unknown cancer stage, among colorectal cancer (CRC) patients with an outpatient or inpatient history of a SPI compared to those with no history of mental illness.

## Methods

We conducted a cross-sectional study designed to examine the relationship between a SPI history and unknown cancer stage at diagnosis using linked administrative data. CRC patients diagnosed between 01/04/2007 and 31/12/2012 were identified in the Ontario Cancer Registry (OCR) using ICD-9153 and 154. The OCR captures 98% of cancer diagnoses in the province [14, 15]. Individuals were excluded for the following reasons: simultaneous colon and rectum tumour presentation, age < 18 years at diagnosis, previous cancer history, diagnosed on death certificate only, no history of SPI and < 6 months of health insurance eligibility prior to the cancer diagnosis. Individuals who had a history of mental illness but did not meet the definition of a SPI were excluded. Ethics approval for this study was granted by the Health Sciences Research Ethics Board at Queen’s University.

We used provincial administrative databases at ICES (formerly called the Institute for Clinical Evaluative Sciences) in Ontario. ICES houses data on all publicly funded healthcare interactions, including psychiatric and cancer care. The following databases were accessed to measure a severe psychiatric illness history, covariates, cancer descriptors and TNM stage: the Canadian Institute for Health Information-Discharge Abstract Database (CIHI-DAD), ICES Physician Database (IPDB), the Ontario Mental Health Reporting System (OMHRS), the Ontario Health Insurance Plan database (OHIP), the OCR, the National Ambulatory Care Reporting System (NACRS), and the Registered Persons Database (RPDB). The CIHI-DAD and OMHRS contain details on all psychiatric hospital admissions in the province, including ICD-10 (CIHI-DAD) and DSM-IV (OMHRS) diagnostic information. The OHIP database (physician billing data) and IPDB (physician specialty information) were linked to identify psychiatry visits associated with an ICD-9 mental disorders diagnoses. The NACRS contains information on all emergency department visits in the province and each visit is associated with multiple diagnoses (ICD-10). Psychiatric hospitalizations, psychiatrist visits, and psychiatric emergency department visits were used to determine the severe psychiatric illness history. Cancer staging and diagnosis information were determined using data collected by Cancer Care Ontario (CCO), the provincial body responsible for the OCR and provincial cancer care.

SPI status was ascertained from psychiatric hospitalizations, psychiatry visits, and psychiatric emergency department visits for diagnoses of major depression, bipolar disorder, schizophrenia, and other psychotic illnesses in the 6 months to 5 years preceding the cancer diagnosis [16]. Individuals with an inpatient and outpatient SPI history were studied separately to capture heterogeneity in mental illness severity. This is in line with recommendations for measuring SPI in the absence of functional status and disability data [17, 18]. AJCC/UICC TNM cancer stage was available in the OCR and collected in the peri-diagnostic period through the Collaborative Staging System© and through the OCR’s contact with regional cancer centres (RCCs) [19]. Best stage is assigned by the OCR. When data from Collaborative Staging is available, it constitutes the best stage. When these data are not available, staging data from the RCC is input. Individuals with a best stage in Stage 0 to Stage IV were considered staged. Individuals missing TNM stage or with a best stage coded as Unknown or Null were considered unknown stage.

Age at diagnosis and sex were obtained from the OCR. Rurality was estimated using the Rural Index of Ontario (RIO) score from data housed in the RPDB. The RIO score was developed as a continuous measure to reflect relative differences in geographic isolation that may impact health and healthcare [20, 21]. However, the RIO should not be included as a continuous variable as unit differences in the RIO score are not equal distances apart. Physical co-morbidities were measured from hospitalization, emergency department, and physician billing data in the six to 18 months prior to the cancer diagnosis using the 32 John’s Hopkins Aggregate Diagnosis Groups (ADGs) [22]. Six ADGs were classified as ‘Major’ physical ADGs and 22 ADGs were classified as ‘Minor’ physical ADGs based on information on the type, diagnosis, and number of encounters and interventions [22]. Quintiles for the four dimensions of the Ontario Marginalization Index (community residential instability, material deprivation, dependency, and ethnic concentration) were measured from Census data linked to postal code [23, 24] and used as proxy measures for individual level marginalization.

Additional information at diagnosis, such as primary tumour site, histology, year of diagnosis, best source of diagnostic information and method diagnosis confirmed were collected at a population-level using established, standard data capture procedures by the OCR from hospital records, regional cancer centre records, emergency department records and death certificates [25]. The best source of diagnostic information during the study time period was defined by CCO as a regional cancer centre and histology as the best method of diagnostic confirmation [25].

### Statistical analysis

Descriptive statistics were presented. Kruskall-Wallis tests compared skewed continuous data and Chi-square tests for independence compared categorical variables. The crude and adjusted relative risks and 95% confidence intervals were estimated using bivariate and multivariable modified Poisson regression with robust error variance. We outlined hypothesized causal pathways and known predictors of unknown stage in our data to identify confounding variables separately from causal pathway variables. Additional file 1: Figures S1-S4 provide detailed directed acyclic graphs of the hypothesized causal relationships. Age (< 45, 45–54, 55–64, 65–74, 75–84, 85+), sex, RIO score (0–9, 10–30, 31–45, 46–55, 56–75, 75+, Unknown), and year of diagnosis were included as covariates in the adjusted analyses. Only RIO score was missing for some patients. Age was modeled as a nominal, categorical variable. RIO scores were categorized according to previously published work. Cell sizes < 6 are suppressed in accordance with ICES privacy and confidentiality requirements. Robustness of the findings was evaluated by re-analyzing the relationship between an SPI history and unknown stage using modified SPI definitions according to known properties of disease algorithms designed for administrative data (e.g., two-year timeframe to evaluate SPI history, increased the minimum number of psychiatrist and/or ED visits to identify a positive SPI history, included family physician visits with SPI-related diagnoses in identifying a positive SPI history).

## Results

Forty-two thousand five hundred ten patients with CRC met the inclusion criteria. The final study cohort included 24,507 CRC patients: 288 were excluded with a simultaneous colon and rectum tumour, 10 were under 18 years old at diagnosis, 4000 had a history of cancer, 107 were diagnosed on death certificate only, 307 were missing exposure information and 13,291 had an “other” mental health history. An SPI history was documented in 740 (3.0%) of patients, 258 (1.1%) had ≥1 psychiatric hospitalization and 482 (2.0%) had ≥1 psychiatrist or emergency department visit only. The distribution of demographic factors (e.g. age, sex, physical comorbidity), clinical factors (e.g., tumour location), and residential factors (e.g., degree of residential rurality, marginalization) varied according to SPI status (Table 1).

**Table 1:** Demographic, clinical, and routinely cancer diagnosis details collected for all CRC patients, stratified by SPI status (column percentages reported)

	No History of Mental Illness (***n*** = 23,767)	Outpatient SPI History (***n*** = 482)	Inpatient SPI History (***n*** = 258)	***P***-value
**Demographic and clinical details**				
**Age at diagnosis (years)**				< 0.001
< 45	3.8	5.4	6.2	
45–54	10.9	**16.2**	14.3	
55–64	22.0	**31.7**	27.5	
65–74	28.3	22.4	25.6	
75–84	25.2	**15.6**	20.9	
≥ 85	9.8	8.7	5.4	
**Sex**				< 0.001
Female	41.0	**50.8**	**53.5**	
**Major Physical Comorbidity**				< 0.001
0 ADGs	64.3	**49.8**	**45.7**	
1 ADGs	26.6	28.0	31.4	
2 ADGs	6.9	**15.4**	**13.6**	
3–6 ADGs	2.2	**6.8**	**9.3**	
**Deprivation**^**e**^				0.003
Least Marginalized	22.8	22.4	18.2	
2	22.8	21.4	17.4	
3	21.4	18.9	18.6	
4	17.9	18.5	22.5	
Most Marginalized	13.6	17.2	**20.9**	
**Rurality (RIO category)**				< 0.001
0–9 (least rural)	62.9	72.2	65.1	
10–30	18.2	15.6	13.6	
31–45	10.3	8.9	10.1	
46–55	2.9	**–**	3.1	
56–75	3.0	2.3	3.9	
> 75 (most rural)	1.3	0.0	–	
Unknown	1.3	–	–	
**Diagnosis and staging details**				
**Diagnosis confirmation**				< 0.001
Histology	93.5	91.1	86.4	
Operation/Other Unknown^a^	6.4	9.0	**13.6**	
**Best source of diagnostic information**				< 0.001
RCC	62.8	60.6	49.6	
Pathology	31.9	32.2	38.8	
Hospital/Inpatient Record/ Unknown^b^	5.4	7.3	**11.6**	
**Histology**				< 0.001
No histology/Unspecified	5.4	7.7	**12.0**	
**Tumour Location**				0.38
NOS/Other^c^	10.9	12.4	12.8	
**TNM Stage**^d^				< 0.001
Stage 0/I	20.0	22.2	18.2	
Stage II	23.2	23.0	20.9	
Stage III	26.1	22.2	23.6	
Stage IV	17.6	16.8	17.4	
Unknown Stage	13.1	15.8	19.8	

^a^Other/Unknown were combined due to cell sizes of ≤1% in both, reported combined with Operation due to small cell sizes; Other includes autopsy, cytology, judgmental, and x-ray; ^b^Unknown accounts for < 1%, reported combined due to small cell sizes; ^c^Other tumour location is < 1%; ^d^Stage 0 was combined with stage I due to cell sizes < 1%; ^e^Data available on 24,155 CRC patients; SPI = severe psychiatric illness; Kruskall-Wallis tests for skewed continuous data and Chi-square tests for independence for categorical variables were used to investigate the relationship between severe psychiatric illness history status and demographic and cancer characteristics. Cells with Pearson residual values ≥3 contributed most significantly to the lack of independence between demographic characteristics and an SPI history and are highlighted with bold font type

Significantly fewer (42%) CRC patients with an inpatient SPI history had complete, high quality data on all routinely collected cancer diagnosis variables (tumour location, histology, confirmation of diagnosis, best source of diagnostic information), compared to 56% of patients with an outpatient SPI history and 59% of patients with no mental illness history (*p* < 0.001) (Table 1). More individuals with an inpatient SPI history had hospital or pathology data as the best source of diagnostic information rather than at an RCC, the gold standard during the time period. A significantly larger proportion of patients with an inpatient SPI history had their CRC diagnosis confirmed using operative records rather than histology, the gold standard, compared to patients with no history of a mental illness.

Stage at diagnosis was unknown for 3120 (13.1%) CRC patients with no history of a mental illness, 76 (15.8%) patients with an outpatient SPI history and 51 (19.8%) of patients with an inpatient SPI history. The proportion of missing stage data among individuals with an inpatient SPI ranged from 20 to 30% across sensitivity analysis definitions. There was a significant difference in the distribution of stage at diagnosis by SPI status (*p* < 0.001). When patients with an unknown stage of cancer were excluded from the analysis (*n* = 4147), the stage distribution no longer varied significantly based on SPI history status (χ^2^ Statistic = 4.0; *p* = 0.68).

After adjusting for confounders, CRC patients with an inpatient SPI history had 1.45 times the risk of a missing TNM stage at diagnosis, compared to patients with no history of a mental illness (95% CI: 1.14–1.85; *p* = 0.01). Patients with an outpatient SPI history had 1.17 times the risk of an unknown cancer stage at diagnosis, compared to patients with no history of mental illness; this risk was marginally significant (95% CI 0.95–1.43). The results did not change when alternate practices to assign SPI history using administrative data were used.

## Discussion

This study suggests potential cancer care inequalities exist in the diagnosis and staging of individuals with an SPI history and CRC; the underlying mechanisms of which may be generalizable to other cancers. Our findings are consistent with the hypothesis that patients with cancer who also experience vulnerable circumstances, such as poverty, older age, or complex health status, have a greater risk of incomplete cancer staging [26–32]. This study provides further evidence that missing stage data are not only the function of data collection processes or quality control issues. Multiple pathways involving poor access to healthcare, medical contraindications, and a lack of patient-centered care exist.

We made many efforts to address the limitations inherent in our study using routinely collected healthcare data. Identifying mental health diagnoses in administrative data is difficult and subject to error [11]. We took numerous steps to ensure a valid comparison. We created a clean reference group by excluding individuals with any record of mental health service use from those who did not meet our definition of a severe psychiatric illness. This would reduce the likelihood of misclassification. Our conclusions did not change after performing multiple sensitivity analyses. This study was cross-sectional as a function of how cancer patients are identified in the registry data, and when staging occurs relative to diagnosis. We enhanced the study design by using a 6 month lag period to begin collecting information from mental healthcare encounters collected separately from the cancer diagnostic process to determine SPI history. This established temporality and reduced the possibility of reverse causality. Incorrect conceptualization of the causal pathway may also result in residual confounding of the observed association. However, existing literature supports our causal pathway hypothesis [4] and our use of established methods [33] to create the causal diagram support the analytic decisions. It is possible that many unstaged patients were frankly metastatic and so although staging investigations were not performed, clinical stage data not collected by the registry were available to the oncologist and the patient to make treatment decisions. However, if that was the case, the exclusion of these patients from studies investigating the association between stage and an SPI history would still be biased, as these data would be missing not at random.

## Conclusion

CRC patients with an SPI history had significantly more cancer staging details missing than patients with no history of mental illness. Patients with an inpatient SPI history were 55% more likely to be missing a TNM stage at diagnosis than patients with no mental illness history (RR 1.45, 1.14–1.85). The absence of high quality diagnostic and staging information has serious consequences. Staging data guide the type and intensity of cancer care and if the process does not occur completely it may interfere with patient outcomes. In our study, appropriate patient care may have still been provided to patients without a TNM stage recorded in the provincial registry if staging data were available clinically or if the patient and their support system agreed it was inappropriate to undergo invasive staging investigations. However, the extent of missing data in the registry may result in systematic bias and inaccurate conclusions in research studies examining cancer care disparities for individuals with an SPI history [11], particularly if patients with missing data are excluded. The inadvertent, yet systematic exclusion of many patients with an SPI history from cancer outcomes research, as the result of missing or unavailable diagnostic or staging data, may also influence generalizability. It is important that when performing these standard exclusions, researchers and clinicians understand not only the methodological implications, but also the clinical implications for rendering these vulnerable populations invisible.

## Supplementary information

**Additional file 1.**

## Data Availability

The data set from this study is held securely in coded form at ICES. While data sharing agreements prohibit ICES from making the data set publicly available, access may be granted to those who meet pre-specified criteria for confidential access, available at www.ices.on.ca/DAS. The full data set creation plan and underlying analytic code are available from the authors upon request, understanding that the programs may rely upon coding templates or macros that are unique to ICES.

## Abbreviations

AJCC: American Joint Committee on Cancer
ADG: Aggregate Diagnosis Groups
CCO: Cancer Care Ontario
CRC: Colorectal cancer
CIHI-DAD: Canadian Institute of Health Information Discharge Abstract Database
DSM: Diagnostic and Statistical Manual
ED: Emergency department
ICD: International Classification of Disease
NACRS: National Ambulatory Care Reporting System
OCR: Ontario Cancer Registry
OHIP: Ontario Health Insurance Plan
OMHRS: Ontario Mental Health Reporting System
RIO: Rurality Index of Ontario
RPDB: Registered Persons Database
SPI: Severe psychiatric illness
TNM: Tumour, lymp node, metastasis
UICC: Union International Cancer Control
