# Supplementary material for: Cancer staging in individuals with a severe psychiatric illness: a cross-sectional study using population-based cancer registry data
Source: BMC Cancer. 2020 May 27;20:476. doi: 10.1186/s12885-020-06943-w (PMC7251666; doi:10.1186/s12885-020-06943-w)
Supplement: Supplementary file 1 — Additional file 1. [file 12885_2020_6943_MOESM1_ESM.docx]

**Supplementary Appendix: Causal pathways from a severe psychiatric illness to unknown stage at diagnosis in individuals with cancer ^1^**

We reviewed the literature to identify potential confounders of the relationship between a severe psychiatric illness history and unknown cancer stage. Based on our literature review we identified two possible pathways to an unknown TNM stage at diagnosis: 1) TNM stage was truly known but the information is not successfully collected from the medical charts and electronic health records by the cancer registry (Figure 1); and 2) TNM stage was truly unknown because it was not clinically ascertained (Figure 2). We hypothesized that a severe psychiatric illness has a causal association with a truly unknown TNM stage via the second pathway.

Based on this hypothesis, we created a directed acyclic graph to identify potential confounders of this relationship reported a truncated version showing only potentially causal relationships. These are represented with arrows from a severe psychiatric illness to the intermediate variable to unknown stage (Figure 3). The total effect of a severe psychiatric illness history is considered the sum of all arrows going from a severe psychiatric illness to unknown stage. We removed all variables directly on the hypothesized causal pathway (Path 2) and included the known stage pathway variables (Path 1) to identify potentially confounding relationships (Figure 5). In Figure 4 the dotted line represents a strong predictor of the outcome via the known pathway that may confound the association under study.


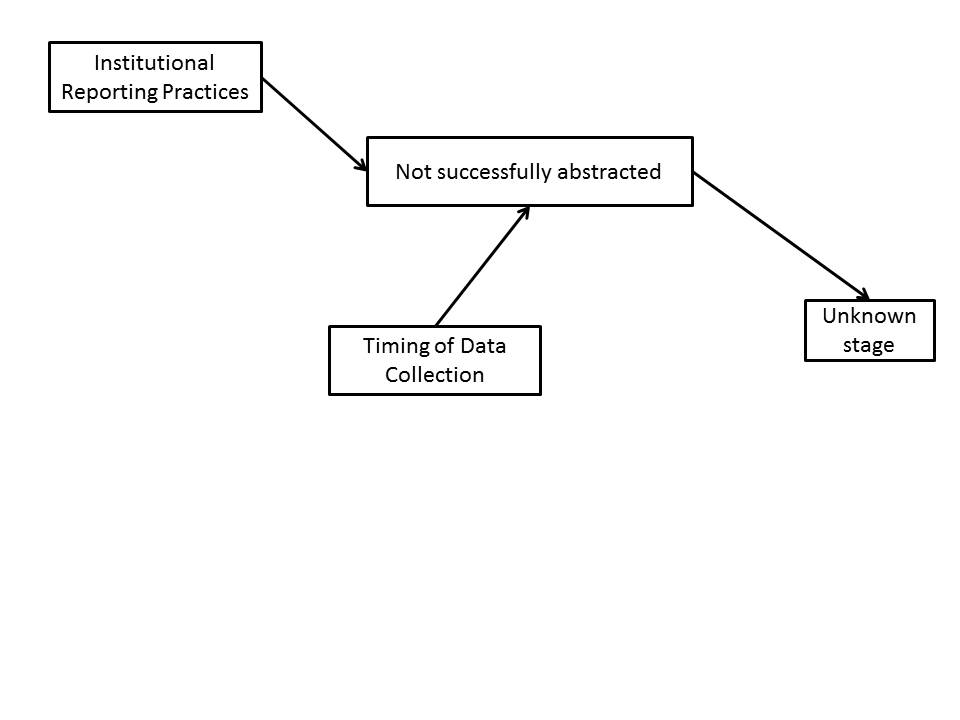


Figure 1 Pathway to unknown stage when a TNM stage is known but not successfully abstracted


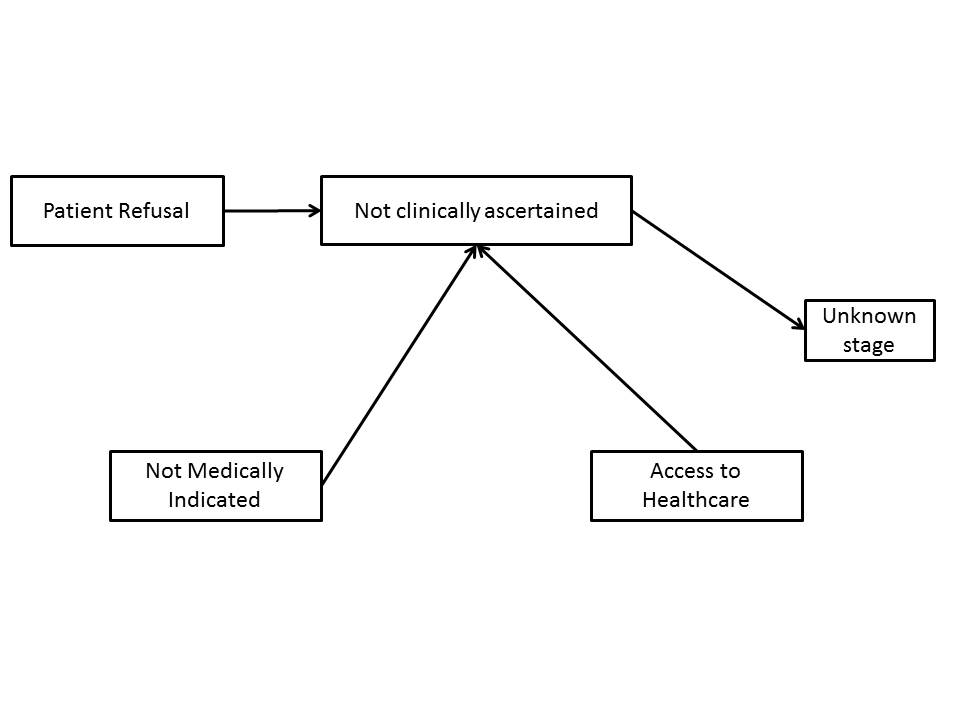


Figure 2 Pathway to unknown stage where TNM stage is never clinically ascertained


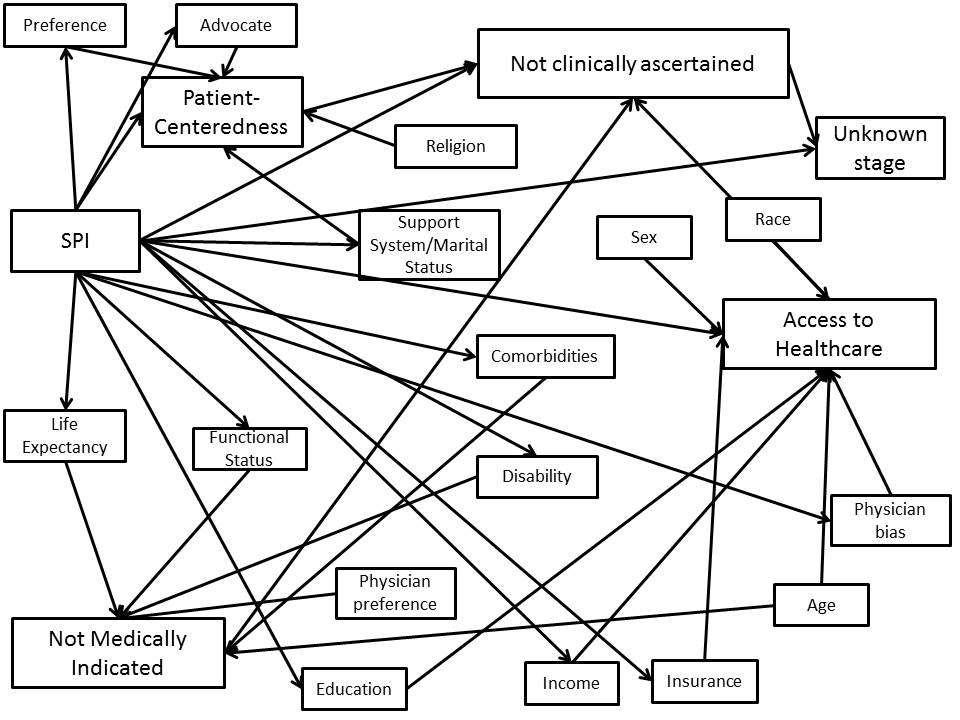


Figure 3 Pathways from a severe psychiatric illness history to an unknown TNM cancer stage when the stage was not clinically ascertained


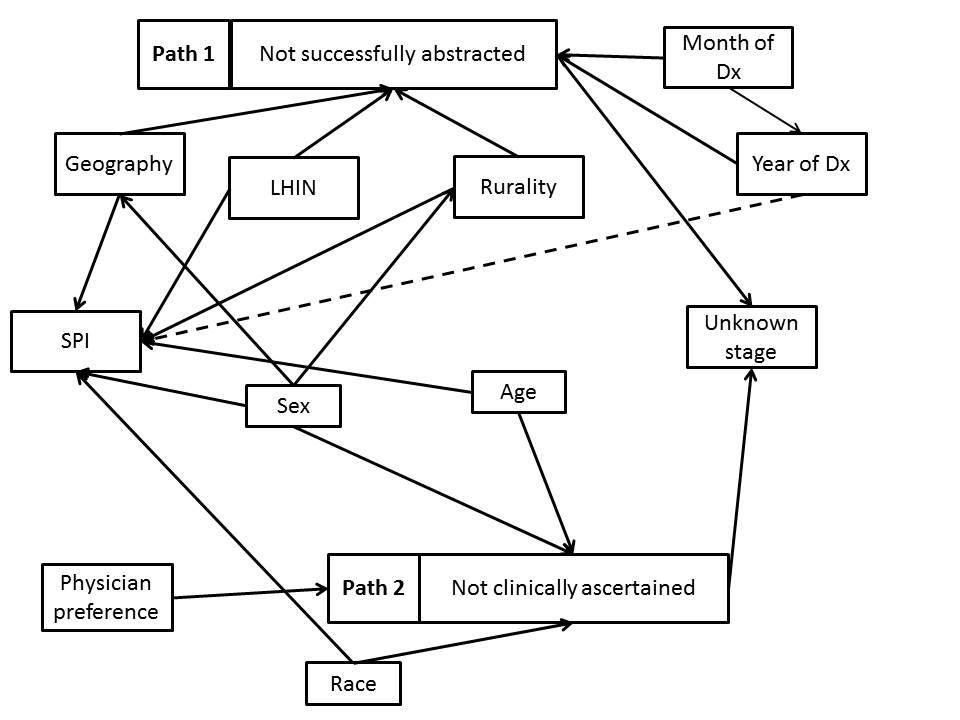


Figure 4 Potential backdoor pathways from a severe psychiatric illness history to an unknown TNM stage through both hypothesized mechanisms

**References**

1. Mahar A. *The impact of a severe psychiatric illness on a cancer diagnosis, treatment, and survival*. Kingston, Ontario: Department of Community Health Sciences, Queen's University; 2017.
